# Poly[[diaqua­[μ_2_-3-carb­oxy-5-(pyridine-4-carboxamido)­benzoato][μ_4_-5-(pyridine-4-carboxamido)­isophthalato]cerium(III)] monohydrate]

**DOI:** 10.1107/S1600536812013402

**Published:** 2012-04-04

**Authors:** Yi-Fang Deng, Xue Nie

**Affiliations:** a Department of Chemistry and Materials Science, Hengyang Normal University, Hengyang 421008, People’s Republic of China

## Abstract

In the title compound, {[Ce(C_14_H_9_N_2_O_5_)(C_14_H_8_N_2_O_5_)(H_2_O)_2_]·H_2_O}_*n*_, three carboxyl groups of two independent isophthalate anions are deprotonated and they bridge the Ce^III^ cations, forming a two-dimensional polymeric structure parallel to (001); another carboxyl group is not deprotonated and links with the adjacent pyridine ring *via* an O—H⋯N hydrogen bond. The Ce^III^ cation is coordinated by six O atoms from carboxyl groups and two O atoms from coordinated water mol­ecules in a distorted square-anti­prismatic arrangement. Extensive O—H⋯O and O—H⋯N hydrogen bonding occurs in the crystal structure.

## Related literature

For applications of lanthanide complexes with carboxyl ligands, see: Chin *et al.* (1994 ▶); Singh *et al.* (2002 ▶). For related complexes, see: Chen *et al.* (2011 ▶); Deng (2011 ▶); Qiu *et al.* (2007 ▶); Gubina *et al.* (2000 ▶); Wang *et al.* (2003 ▶).
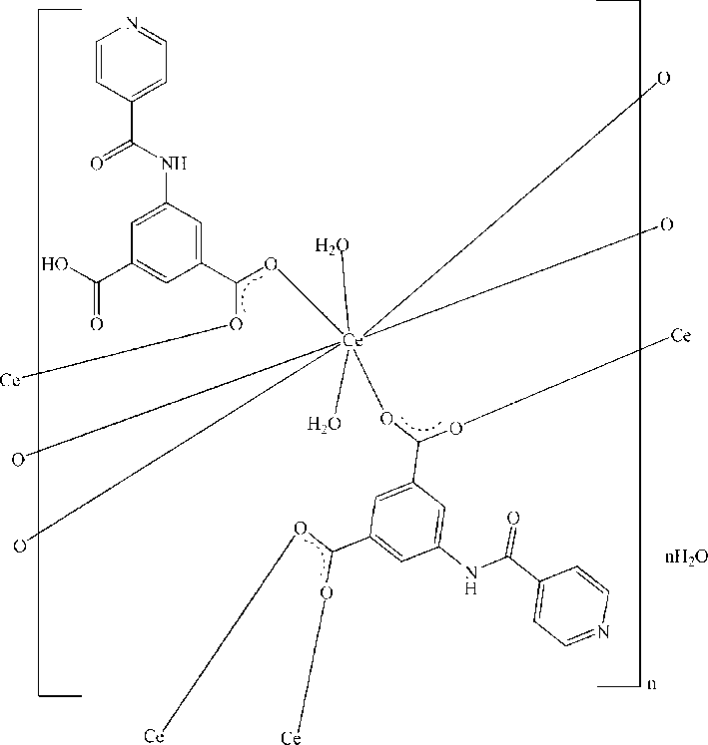


## Experimental

### 

#### Crystal data

[Ce(C_14_H_9_N_2_O_5_)(C_14_H_8_N_2_O_5_)(H_2_O)_2_]·H_2_O
*M* *_r_* = 763.62
Triclinic, 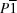

*a* = 9.6742 (8) Å
*b* = 10.6187 (8) Å
*c* = 15.8542 (12) Å
α = 81.443 (1)°
β = 78.753 (2)°
γ = 64.166 (2)°
*V* = 1433.98 (19) Å^3^
*Z* = 2
Mo *K*α radiation
μ = 1.67 mm^−1^
*T* = 293 K
0.18 × 0.14 × 0.08 mm

#### Data collection

Bruker APEXII CCD diffractometer
Absorption correction: multi-scan (*SADABS*; Bruker, 2001 ▶) *T*_min_ = 0.754, *T*_max_ = 0.878
7189 measured reflections
4972 independent reflections
4669 reflections with *I* > 2σ(*I*)
*R*_int_ = 0.071

#### Refinement

*R*[*F* ^2^ > 2σ(*F* ^2^)] = 0.033
*wR*(*F* ^2^) = 0.086
*S* = 1.04
4972 reflections
416 parameters
H-atom parameters constrained
Δρ_max_ = 1.26 e Å^−3^
Δρ_min_ = −1.23 e Å^−3^

### 

Data collection: *APEX2* (Bruker, 2007 ▶); cell refinement: *SAINT* (Bruker, 2007 ▶); data reduction: *SAINT*; program(s) used to solve structure: *SHELXTL* (Sheldrick, 2008 ▶); program(s) used to refine structure: *SHELXTL*; molecular graphics: *SHELXTL*; software used to prepare material for publication: *SHELXTL*.

## Supplementary Material

Crystal structure: contains datablock(s) global, I. DOI: 10.1107/S1600536812013402/xu5485sup1.cif

Structure factors: contains datablock(s) I. DOI: 10.1107/S1600536812013402/xu5485Isup2.hkl

Additional supplementary materials:  crystallographic information; 3D view; checkCIF report

## Figures and Tables

**Table 1:** Hydrogen-bond geometry (Å, °)

*D*—H⋯*A*	*D*—H	H⋯*A*	*D*⋯*A*	*D*—H⋯*A*
O1*W*—H1*WA*⋯N2^i^	0.85	2.21	2.756 (5)	122
O2*W*—H2*WB*⋯O1*W*^ii^	0.85	2.53	3.297 (5)	150
O2*W*—H2*WA*⋯O3*W*^iii^	0.85	2.07	2.728 (6)	133
O3*W*—H3*WA*⋯O10^iv^	0.85	2.06	2.856 (8)	157
O3*W*—H3*WB*⋯O5^v^	0.85	1.92	2.742 (6)	161
O3—H3⋯N4^vi^	0.82	1.81	2.583 (5)	156

Symmetry codes: (i) 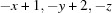; (ii) 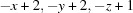; (iii) 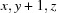; (iv) 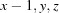; (v) 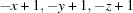; (vi) 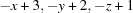.

**Fig. 1.**
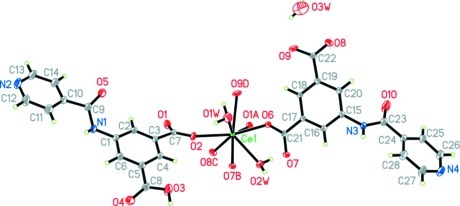
The ORTEP drawing of the title compound (I). Displacement ellipsoids are drawn at 30% probability level. [Symmetry codes: (i) 1 - x,2 - y,1 - z (ii) 2 - x,2 - y,1 - z (iii) -1 + x,1 + y,z (iv) 2 - x,1 - y,1 - z.]

**Fig. 2.**
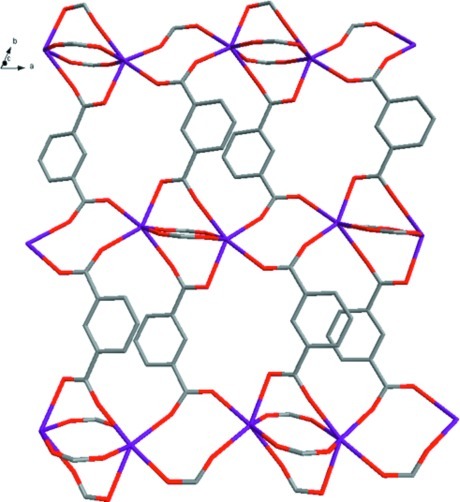
Projection showing the two-dimensional structure of the compound, all pyridyl groups are omitted for clarity.

## supplementary crystallographic information

### Comment

Recently, lanthanide-carboxylic acid complexes have been widely studied and applied in many fields due to their excellent luminescent properties (Chin *et al.*, 1994; Singh *et al.*, 2002). Distinct structure features with various lanthanides (Qiu *et al.*, 2007; Gubina *et al.*, 2000) or ligands (Wang *et al.*, 2003) have been reported.

The title compound, (I) was synthesized and its structure was determined by X-ray diffraction. Similar crystal structure with 5-isonicotinamidoisophthalic acid as ligand has been reported recently (Chen *et al.*, 2011; Deng, 2011).

In the title compound, the central Ce^III^ ion is eight-coordinated by two O atoms from two water molecules, two carboxylate O atoms from two partial-deprotonated H*L*^-^ ligands and four other O atoms from four different *L*^2-^ ligands, which forming a distorted square-antiprismatic geometry (Fig. 1). Moreover, the H*L*^-^ anions adopt µ_2_-η^1^:η^1^ bridging coordination mode, while two carboxylate groups of each *L*^2-^ ligand have different coordination modes, one is µ_2_-η^1^:η^1^ bridging and the other one acts as µ_2_-η^2^:η^1^-bridging coordination mode, whereas the pyridyl group is free of coordination. Such a coordination mode makes (I) into an infinite two-dimensional network (Fig. 2). The pyridyl groups are free. Adjacent molecules are linked through O—H···N and O—H···O hydrogen bonds into a three-dimensional network.

### Experimental

A mixture of 0.05 mmol Ce(NO_3_)_3_.6H_2_O (21.2 mg. 0.05 mmol), 5-isonicotinamidoisophthalic acid (28.7 mg, 0.1 mmol), NaOH (6.0 mg, 0.15 mmol), MeOH (5 ml) and water (5 ml) was heated in a 16 ml capacity Teflon-lined reaction vessel at 453 K for 3 d, the reaction mixture then was cooled to room temperature over a period of 40 h. The product was collected by filtration.

### Refinement

Amide H atoms were located in a difference Fourier map and refined as riding in as found relative positions, other H atoms were placed geometrically with O—H = 0.86 (water), 0.82 (carboxyl) and C—H = 0.93 Å), and refined in riding mode, *U*_iso_(H) = 1.2*U*_eq_(N,*O*,*C*).

### Crystal data

**Table:**

[Ce(C_14_H_9_N_2_O_5_)(C_14_H_8_N_2_O_5_)(H_2_O)_2_]·H_2_O	*Z* = 2
*M**_r_* = 763.62	*F*(000) = 762
Triclinic, *P*1	*D*_x_ = 1.769 Mg m^−^^3^
Hall symbol: -P 1	Mo *K*α radiation, λ = 0.71073 Å
*a* = 9.6742 (8) Å	Cell parameters from 4972 reflections
*b* = 10.6187 (8) Å	θ = 2.1–25.0°
*c* = 15.8542 (12) Å	µ = 1.67 mm^−^^1^
α = 81.443 (1)°	*T* = 293 K
β = 78.753 (2)°	Block, colorless
γ = 64.166 (2)°	0.18 × 0.14 × 0.08 mm
*V* = 1433.98 (19) Å^3^	

### Data collection

**Table:**

Bruker APEXII CCD diffractometer	4972 independent reflections
Radiation source: fine-focus sealed tube	4669 reflections with *I* > 2σ(*I*)
Graphite monochromator	*R*_int_ = 0.071
φ and ω scans	θ_max_ = 25.0°, θ_min_ = 2.1°
Absorption correction: multi-scan (*SADABS*; Bruker, 2001)	*h* = −11→11
*T*_min_ = 0.754, *T*_max_ = 0.878	*k* = −10→12
7189 measured reflections	*l* = −16→18

### Refinement

**Table:**

Refinement on *F*^2^	Primary atom site location: structure-invariant direct methods
Least-squares matrix: full	Secondary atom site location: difference Fourier map
*R*[*F*^2^ > 2σ(*F*^2^)] = 0.033	Hydrogen site location: inferred from neighbouring sites
*wR*(*F*^2^) = 0.086	H-atom parameters constrained
*S* = 1.04	*w* = 1/[σ^2^(*F*_o_^2^) + (0.0565*P*)^2^] where *P* = (*F*_o_^2^ + 2*F*_c_^2^)/3
4972 reflections	(Δ/σ)_max_ = 0.002
416 parameters	Δρ_max_ = 1.26 e Å^−^^3^
0 restraints	Δρ_min_ = −1.23 e Å^−^^3^

### Special details

**Table:**

Geometry. All e.s.d.'s (except the e.s.d. in the dihedral angle between two l.s. planes) are estimated using the full covariance matrix. The cell e.s.d.'s are taken into account individually in the estimation of e.s.d.'s in distances, angles and torsion angles; correlations between e.s.d.'s in cell parameters are only used when they are defined by crystal symmetry. An approximate (isotropic) treatment of cell e.s.d.'s is used for estimating e.s.d.'s involving l.s. planes.
Refinement. Refinement of *F*^2^ against ALL reflections. The weighted *R*-factor *wR* and goodness of fit *S* are based on *F*^2^, conventional *R*-factors *R* are based on *F*, with *F* set to zero for negative *F*^2^. The threshold expression of *F*^2^ > σ(*F*^2^) is used only for calculating *R*-factors(gt) *etc*. and is not relevant to the choice of reflections for refinement. *R*-factors based on *F*^2^ are statistically about twice as large as those based on *F*, and *R*- factors based on ALL data will be even larger.

### Fractional atomic coordinates and isotropic or equivalent isotropic displacement parameters (Å^2^)

**Table:**

	*x*	*y*	*z*	*U*_iso_*/*U*_eq_	
Ce1	0.731679 (19)	0.960889 (17)	0.503370 (11)	0.01875 (9)	
C7	0.5111 (4)	1.0993 (4)	0.3253 (2)	0.0249 (7)	
C3	0.5318 (4)	1.1620 (4)	0.2339 (2)	0.0259 (7)	
C2	0.4389 (4)	1.1697 (4)	0.1751 (2)	0.0288 (8)	
H2	0.3671	1.1309	0.1903	0.035*	
C1	0.4534 (4)	1.2359 (4)	0.0934 (2)	0.0299 (8)	
C6	0.5503 (5)	1.3043 (4)	0.0741 (2)	0.0353 (9)	
H6	0.5531	1.3560	0.0215	0.042*	
C5	0.6427 (5)	1.2962 (4)	0.1326 (2)	0.0323 (8)	
C4	0.6388 (4)	1.2197 (4)	0.2109 (2)	0.0299 (8)	
H4	0.7079	1.2070	0.2481	0.036*	
C9	0.3250 (5)	1.1435 (5)	0.0155 (3)	0.0385 (9)	
C10	0.2671 (5)	1.1588 (4)	−0.0677 (2)	0.0364 (9)	
C14	0.1640 (6)	1.1015 (5)	−0.0707 (3)	0.0480 (11)	
H14	0.1281	1.0590	−0.0213	0.058*	
C13	0.1153 (6)	1.1087 (6)	−0.1486 (3)	0.0575 (13)	
H13	0.0458	1.0705	−0.1502	0.069*	
C12	0.2624 (7)	1.2209 (6)	−0.2179 (3)	0.0562 (13)	
H12	0.2969	1.2615	−0.2687	0.067*	
C11	0.3175 (6)	1.2199 (5)	−0.1436 (3)	0.0496 (11)	
H11	0.3869	1.2593	−0.1445	0.059*	
C8	0.7431 (5)	1.3750 (5)	0.1128 (3)	0.0398 (10)	
C21	1.1064 (4)	0.7882 (3)	0.5569 (2)	0.0212 (7)	
C17	1.2354 (4)	0.6476 (3)	0.5727 (2)	0.0210 (7)	
C16	1.3614 (4)	0.6391 (4)	0.6063 (2)	0.0254 (7)	
H16	1.3649	0.7207	0.6183	0.031*	
C15	1.4816 (4)	0.5108 (4)	0.6220 (2)	0.0267 (7)	
C20	1.4817 (4)	0.3891 (4)	0.6011 (2)	0.0267 (8)	
H20	1.5633	0.3028	0.6113	0.032*	
C19	1.3580 (4)	0.3973 (4)	0.5645 (2)	0.0230 (7)	
C18	1.2335 (4)	0.5254 (3)	0.5521 (2)	0.0223 (7)	
H18	1.1491	0.5296	0.5301	0.027*	
C23	1.6591 (5)	0.4429 (4)	0.7281 (3)	0.0389 (9)	
C24	1.7777 (4)	0.4767 (4)	0.7573 (3)	0.0337 (9)	
C28	1.8537 (5)	0.5516 (5)	0.7061 (3)	0.0429 (10)	
H28	1.8401	0.5784	0.6487	0.052*	
C27	1.9499 (5)	0.5854 (5)	0.7425 (3)	0.0487 (12)	
H27	1.9997	0.6369	0.7083	0.058*	
C26	1.9051 (5)	0.4746 (5)	0.8713 (3)	0.0410 (10)	
H26	1.9236	0.4468	0.9280	0.049*	
C25	1.8059 (5)	0.4367 (5)	0.8415 (3)	0.0388 (10)	
H25	1.7583	0.3848	0.8773	0.047*	
C22	1.3639 (4)	0.2695 (4)	0.5316 (2)	0.0266 (7)	
N1	0.3669 (4)	1.2433 (4)	0.0286 (2)	0.0359 (8)	
H2A	0.4223	1.2574	−0.0215	0.043*	
N2	0.1632 (5)	1.1672 (5)	−0.2205 (3)	0.0572 (11)	
N3	1.6082 (4)	0.5117 (3)	0.6555 (2)	0.0326 (7)	
H3A	1.6550	0.5606	0.6263	0.039*	
N4	1.9759 (4)	0.5488 (4)	0.8236 (2)	0.0443 (9)	
O1	0.3837 (3)	1.0938 (3)	0.35242 (16)	0.0368 (6)	
O2	0.6201 (3)	1.0635 (3)	0.36808 (16)	0.0342 (6)	
O3	0.8519 (5)	1.3290 (5)	0.1605 (3)	0.0757 (13)	
H3	0.8914	1.3846	0.1553	0.114*	
O4	0.7212 (4)	1.4718 (4)	0.0589 (2)	0.0537 (9)	
O5	0.3353 (5)	1.0419 (4)	0.0661 (2)	0.0589 (10)	
O6	0.9822 (3)	0.7910 (2)	0.54280 (17)	0.0303 (6)	
O7	1.1291 (3)	0.8947 (2)	0.56132 (17)	0.0300 (6)	
O8	1.4778 (3)	0.1545 (3)	0.53961 (18)	0.0381 (6)	
O9	1.2553 (4)	0.2827 (3)	0.4946 (3)	0.0585 (10)	
O10	1.6089 (5)	0.3622 (5)	0.7707 (2)	0.0748 (13)	
O1W	0.9238 (4)	0.8370 (3)	0.37514 (18)	0.0537 (8)	
H1WB	0.8907	0.7774	0.3687	0.064*	
H1WA	0.9094	0.8880	0.3282	0.064*	
O2W	0.7983 (3)	1.0672 (3)	0.61536 (17)	0.0369 (6)	
H2WB	0.8768	1.0855	0.5974	0.044*	
H2WA	0.8080	1.0189	0.6634	0.044*	
O3W	0.7921 (9)	0.0666 (7)	0.7882 (3)	0.141 (3)	
H3WA	0.7410	0.1497	0.7680	0.169*	
H3WB	0.7469	0.0249	0.8251	0.169*	

### Atomic displacement parameters (Å^2^)

**Table:**

	*U*^11^	*U*^22^	*U*^33^	*U*^12^	*U*^13^	*U*^23^
Ce1	0.01952 (13)	0.01527 (12)	0.02393 (13)	−0.00835 (9)	−0.00774 (8)	0.00066 (8)
C7	0.0299 (19)	0.0230 (18)	0.0252 (17)	−0.0131 (15)	−0.0082 (14)	0.0005 (14)
C3	0.0283 (19)	0.0244 (18)	0.0276 (18)	−0.0127 (15)	−0.0074 (14)	0.0006 (14)
C2	0.032 (2)	0.032 (2)	0.0277 (18)	−0.0182 (17)	−0.0085 (15)	0.0009 (15)
C1	0.036 (2)	0.032 (2)	0.0289 (18)	−0.0183 (17)	−0.0137 (16)	0.0009 (15)
C6	0.044 (2)	0.040 (2)	0.0303 (19)	−0.026 (2)	−0.0115 (17)	0.0070 (17)
C5	0.038 (2)	0.037 (2)	0.0312 (19)	−0.0241 (18)	−0.0091 (16)	0.0024 (16)
C4	0.034 (2)	0.032 (2)	0.0304 (19)	−0.0181 (17)	−0.0119 (16)	0.0007 (15)
C9	0.047 (2)	0.044 (2)	0.036 (2)	−0.028 (2)	−0.0153 (18)	0.0044 (19)
C10	0.046 (2)	0.038 (2)	0.032 (2)	−0.0217 (19)	−0.0141 (17)	0.0011 (17)
C14	0.058 (3)	0.059 (3)	0.042 (2)	−0.035 (3)	−0.018 (2)	0.000 (2)
C13	0.062 (3)	0.064 (3)	0.058 (3)	−0.027 (3)	−0.026 (3)	−0.016 (3)
C12	0.070 (3)	0.068 (3)	0.032 (2)	−0.027 (3)	−0.016 (2)	−0.001 (2)
C11	0.062 (3)	0.056 (3)	0.040 (2)	−0.034 (3)	−0.012 (2)	0.001 (2)
C8	0.051 (3)	0.051 (3)	0.035 (2)	−0.037 (2)	−0.0121 (19)	0.0044 (19)
C21	0.0236 (17)	0.0231 (17)	0.0196 (16)	−0.0112 (14)	−0.0054 (13)	−0.0020 (13)
C17	0.0207 (17)	0.0190 (17)	0.0262 (17)	−0.0101 (14)	−0.0072 (13)	0.0006 (13)
C16	0.0284 (19)	0.0244 (18)	0.0305 (18)	−0.0159 (15)	−0.0086 (14)	−0.0013 (14)
C15	0.0250 (18)	0.0271 (19)	0.0315 (18)	−0.0121 (15)	−0.0116 (15)	0.0016 (15)
C20	0.0250 (18)	0.0212 (18)	0.0341 (19)	−0.0086 (15)	−0.0094 (15)	0.0008 (14)
C19	0.0227 (17)	0.0216 (17)	0.0273 (17)	−0.0114 (14)	−0.0059 (14)	0.0006 (14)
C18	0.0204 (17)	0.0209 (17)	0.0302 (18)	−0.0115 (14)	−0.0069 (14)	−0.0018 (14)
C23	0.040 (2)	0.040 (2)	0.046 (2)	−0.023 (2)	−0.0188 (19)	0.0057 (19)
C24	0.029 (2)	0.034 (2)	0.042 (2)	−0.0148 (17)	−0.0149 (17)	0.0018 (17)
C28	0.043 (2)	0.060 (3)	0.037 (2)	−0.030 (2)	−0.0140 (18)	0.006 (2)
C27	0.046 (3)	0.071 (3)	0.046 (2)	−0.041 (3)	−0.015 (2)	0.008 (2)
C26	0.039 (2)	0.053 (3)	0.036 (2)	−0.023 (2)	−0.0123 (18)	0.0020 (19)
C25	0.038 (2)	0.045 (2)	0.041 (2)	−0.024 (2)	−0.0171 (18)	0.0103 (19)
C22	0.0270 (19)	0.0203 (18)	0.0343 (19)	−0.0124 (15)	−0.0035 (15)	−0.0009 (14)
N1	0.049 (2)	0.045 (2)	0.0279 (16)	−0.0308 (17)	−0.0168 (15)	0.0070 (14)
N2	0.068 (3)	0.060 (3)	0.042 (2)	−0.014 (2)	−0.026 (2)	−0.0129 (19)
N3	0.0294 (17)	0.0320 (17)	0.0443 (18)	−0.0168 (14)	−0.0195 (14)	0.0048 (14)
N4	0.043 (2)	0.064 (2)	0.0414 (19)	−0.035 (2)	−0.0151 (16)	0.0026 (17)
O1	0.0304 (15)	0.0464 (17)	0.0334 (14)	−0.0189 (13)	−0.0077 (11)	0.0105 (12)
O2	0.0380 (15)	0.0454 (16)	0.0303 (13)	−0.0250 (13)	−0.0175 (12)	0.0055 (12)
O3	0.088 (3)	0.102 (3)	0.083 (3)	−0.080 (3)	−0.057 (2)	0.047 (2)
O4	0.074 (2)	0.061 (2)	0.0481 (18)	−0.0527 (19)	−0.0203 (16)	0.0207 (16)
O5	0.099 (3)	0.059 (2)	0.0472 (18)	−0.057 (2)	−0.0355 (19)	0.0185 (16)
O6	0.0256 (13)	0.0224 (13)	0.0483 (15)	−0.0106 (11)	−0.0172 (12)	−0.0009 (11)
O7	0.0316 (14)	0.0194 (12)	0.0454 (15)	−0.0148 (11)	−0.0152 (12)	0.0045 (11)
O8	0.0426 (17)	0.0220 (14)	0.0433 (16)	−0.0051 (12)	−0.0116 (13)	−0.0035 (11)
O9	0.051 (2)	0.0288 (16)	0.111 (3)	−0.0155 (14)	−0.041 (2)	−0.0168 (17)
O10	0.089 (3)	0.099 (3)	0.076 (2)	−0.076 (3)	−0.051 (2)	0.042 (2)
O1W	0.069 (2)	0.0480 (19)	0.0349 (16)	−0.0105 (17)	−0.0130 (15)	−0.0133 (14)
O2W	0.0388 (16)	0.0416 (16)	0.0379 (15)	−0.0226 (13)	−0.0083 (12)	−0.0033 (12)
O3W	0.230 (8)	0.154 (6)	0.076 (3)	−0.129 (6)	−0.007 (4)	0.011 (4)

### Geometric parameters (Å, º)

**Table:**

Ce1—O1^i^	2.446 (2)	C17—C16	1.385 (5)
Ce1—O2	2.477 (2)	C17—C18	1.393 (5)
Ce1—O6	2.442 (2)	C16—C15	1.379 (5)
Ce1—O7^ii^	2.452 (2)	C16—H16	0.9300
Ce1—O8^iii^	2.447 (3)	C15—C20	1.381 (5)
Ce1—O9^iv^	2.530 (3)	C15—N3	1.431 (4)
Ce1—O1W	2.552 (3)	C20—C19	1.394 (5)
Ce1—O2W	2.553 (3)	C20—H20	0.9300
C7—O1	1.248 (4)	C19—C18	1.387 (5)
C7—O2	1.252 (4)	C19—C22	1.498 (5)
C7—C3	1.517 (5)	C18—H18	0.9300
C3—C4	1.385 (5)	C23—O10	1.220 (5)
C3—C2	1.386 (5)	C23—N3	1.326 (5)
C2—C1	1.392 (5)	C23—C24	1.508 (5)
C2—H2	0.9300	C24—C25	1.382 (5)
C1—C6	1.385 (5)	C24—C28	1.384 (6)
C1—N1	1.419 (4)	C28—C27	1.374 (6)
C6—C5	1.377 (5)	C28—H28	0.9300
C6—H6	0.9300	C27—N4	1.326 (6)
C5—C4	1.383 (5)	C27—H27	0.9300
C5—C8	1.501 (5)	C26—N4	1.324 (6)
C4—H4	0.9300	C26—C25	1.369 (6)
C9—O5	1.224 (5)	C26—H26	0.9300
C9—N1	1.344 (5)	C25—H25	0.9300
C9—C10	1.492 (5)	C22—O9	1.247 (4)
C10—C11	1.386 (6)	C22—O8	1.248 (4)
C10—C14	1.387 (6)	N1—H2A	0.8998
C14—C13	1.386 (6)	N3—H3A	0.8600
C14—H14	0.9300	O1—Ce1^i^	2.446 (2)
C13—N2	1.316 (7)	O3—H3	0.8200
C13—H13	0.9300	O7—Ce1^ii^	2.452 (2)
C12—N2	1.322 (7)	O8—Ce1^v^	2.447 (3)
C12—C11	1.381 (6)	O9—Ce1^iv^	2.530 (3)
C12—H12	0.9300	O1W—H1WB	0.8499
C11—H11	0.9300	O1W—H1WA	0.8495
C8—O4	1.205 (5)	O2W—H2WB	0.8506
C8—O3	1.291 (5)	O2W—H2WA	0.8495
C21—O6	1.252 (4)	O3W—H3WA	0.8496
C21—O7	1.256 (4)	O3W—H3WB	0.8500
C21—C17	1.496 (5)		
			
O6—Ce1—O1^i^	87.00 (9)	O4—C8—O3	124.6 (4)
O6—Ce1—O8^iii^	150.29 (9)	O4—C8—C5	122.7 (4)
O1^i^—Ce1—O8^iii^	71.37 (9)	O3—C8—C5	112.8 (4)
O6—Ce1—O7^ii^	85.08 (8)	O6—C21—O7	124.9 (3)
O1^i^—Ce1—O7^ii^	137.06 (9)	O6—C21—C17	117.5 (3)
O8^iii^—Ce1—O7^ii^	96.89 (9)	O7—C21—C17	117.6 (3)
O6—Ce1—O2	136.56 (9)	C16—C17—C18	119.4 (3)
O1^i^—Ce1—O2	131.89 (9)	C16—C17—C21	119.3 (3)
O8^iii^—Ce1—O2	72.04 (9)	C18—C17—C21	121.3 (3)
O7^ii^—Ce1—O2	77.34 (8)	C15—C16—C17	120.6 (3)
O6—Ce1—O9^iv^	71.67 (9)	C15—C16—H16	119.7
O1^i^—Ce1—O9^iv^	72.84 (12)	C17—C16—H16	119.7
O8^iii^—Ce1—O9^iv^	118.35 (10)	C16—C15—C20	120.5 (3)
O7^ii^—Ce1—O9^iv^	141.86 (11)	C16—C15—N3	117.0 (3)
O2—Ce1—O9^iv^	98.94 (10)	C20—C15—N3	122.4 (3)
O6—Ce1—O1W	66.88 (9)	C15—C20—C19	119.2 (3)
O1^i^—Ce1—O1W	140.12 (10)	C15—C20—H20	120.4
O8^iii^—Ce1—O1W	141.96 (9)	C19—C20—H20	120.4
O7^ii^—Ce1—O1W	72.58 (10)	C18—C19—C20	120.4 (3)
O2—Ce1—O1W	70.00 (10)	C18—C19—C22	119.1 (3)
O9^iv^—Ce1—O1W	70.61 (12)	C20—C19—C22	120.3 (3)
O6—Ce1—O2W	72.59 (9)	C19—C18—C17	119.8 (3)
O1^i^—Ce1—O2W	70.31 (9)	C19—C18—H18	120.1
O8^iii^—Ce1—O2W	80.89 (9)	C17—C18—H18	120.1
O7^ii^—Ce1—O2W	67.04 (9)	O10—C23—N3	122.6 (4)
O2—Ce1—O2W	131.79 (9)	O10—C23—C24	121.9 (4)
O9^iv^—Ce1—O2W	129.14 (10)	N3—C23—C24	115.5 (3)
O1W—Ce1—O2W	124.10 (10)	C25—C24—C28	118.2 (4)
O1—C7—O2	125.7 (3)	C25—C24—C23	117.8 (4)
O1—C7—C3	116.8 (3)	C28—C24—C23	123.9 (3)
O2—C7—C3	117.4 (3)	C27—C28—C24	118.2 (4)
C4—C3—C2	119.9 (3)	C27—C28—H28	120.9
C4—C3—C7	119.1 (3)	C24—C28—H28	120.9
C2—C3—C7	120.8 (3)	N4—C27—C28	123.8 (4)
C3—C2—C1	119.7 (3)	N4—C27—H27	118.1
C3—C2—H2	120.1	C28—C27—H27	118.1
C1—C2—H2	120.1	N4—C26—C25	123.4 (4)
C6—C1—C2	119.6 (3)	N4—C26—H26	118.3
C6—C1—N1	117.3 (3)	C25—C26—H26	118.3
C2—C1—N1	123.1 (3)	C26—C25—C24	119.0 (4)
C5—C6—C1	120.2 (4)	C26—C25—H25	120.5
C5—C6—H6	119.9	C24—C25—H25	120.5
C1—C6—H6	119.9	O9—C22—O8	121.8 (3)
C6—C5—C4	120.1 (3)	O9—C22—C19	118.4 (3)
C6—C5—C8	120.1 (3)	O8—C22—C19	119.8 (3)
C4—C5—C8	119.8 (3)	C9—N1—C1	125.9 (3)
C5—C4—C3	119.9 (3)	C9—N1—H2A	105.5
C5—C4—H4	120.0	C1—N1—H2A	105.1
C3—C4—H4	120.0	C13—N2—C12	118.3 (4)
O5—C9—N1	124.0 (4)	C23—N3—C15	125.0 (3)
O5—C9—C10	119.6 (4)	C23—N3—H3A	117.5
N1—C9—C10	116.4 (3)	C15—N3—H3A	117.5
C11—C10—C14	118.0 (4)	C26—N4—C27	117.4 (4)
C11—C10—C9	123.6 (4)	C7—O1—Ce1^i^	131.8 (2)
C14—C10—C9	118.3 (4)	C7—O2—Ce1	149.8 (2)
C13—C14—C10	118.9 (5)	C8—O3—H3	109.5
C13—C14—H14	120.5	C21—O6—Ce1	139.3 (2)
C10—C14—H14	120.5	C21—O7—Ce1^ii^	147.2 (2)
N2—C13—C14	122.8 (5)	C22—O8—Ce1^v^	159.1 (3)
N2—C13—H13	118.6	C22—O9—Ce1^iv^	106.3 (2)
C14—C13—H13	118.6	Ce1—O1W—H1WB	101.1
N2—C12—C11	123.5 (5)	Ce1—O1W—H1WA	112.4
N2—C12—H12	118.3	H1WB—O1W—H1WA	101.8
C11—C12—H12	118.3	Ce1—O2W—H2WB	113.4
C12—C11—C10	118.5 (5)	Ce1—O2W—H2WA	113.3
C12—C11—H11	120.8	H2WB—O2W—H2WA	110.7
C10—C11—H11	120.8	H3WA—O3W—H3WB	120.4
			
O1—C7—C3—C4	155.7 (4)	N3—C23—C24—C28	−12.3 (6)
O2—C7—C3—C4	−21.2 (5)	C25—C24—C28—C27	−1.7 (7)
O1—C7—C3—C2	−20.4 (5)	C23—C24—C28—C27	174.8 (4)
O2—C7—C3—C2	162.7 (3)	C24—C28—C27—N4	0.9 (8)
C4—C3—C2—C1	−0.2 (6)	N4—C26—C25—C24	0.3 (7)
C7—C3—C2—C1	175.9 (3)	C28—C24—C25—C26	1.2 (6)
C3—C2—C1—C6	−5.8 (6)	C23—C24—C25—C26	−175.5 (4)
C3—C2—C1—N1	177.5 (4)	C18—C19—C22—O9	−0.5 (5)
C2—C1—C6—C5	6.0 (6)	C20—C19—C22—O9	−175.7 (4)
N1—C1—C6—C5	−177.1 (4)	C18—C19—C22—O8	177.5 (3)
C1—C6—C5—C4	−0.1 (6)	C20—C19—C22—O8	2.3 (5)
C1—C6—C5—C8	−177.7 (4)	O5—C9—N1—C1	11.9 (7)
C6—C5—C4—C3	−5.9 (6)	C10—C9—N1—C1	−166.3 (4)
C8—C5—C4—C3	171.6 (4)	C6—C1—N1—C9	148.1 (4)
C2—C3—C4—C5	6.0 (6)	C2—C1—N1—C9	−35.2 (6)
C7—C3—C4—C5	−170.1 (3)	C14—C13—N2—C12	0.2 (8)
O5—C9—C10—C11	−147.1 (5)	C11—C12—N2—C13	−0.5 (8)
N1—C9—C10—C11	31.2 (7)	O10—C23—N3—C15	5.8 (7)
O5—C9—C10—C14	29.0 (7)	C24—C23—N3—C15	−171.2 (3)
N1—C9—C10—C14	−152.7 (4)	C16—C15—N3—C23	123.6 (4)
C11—C10—C14—C13	−0.4 (7)	C20—C15—N3—C23	−60.1 (5)
C9—C10—C14—C13	−176.8 (5)	C25—C26—N4—C27	−1.1 (7)
C10—C14—C13—N2	0.3 (8)	C28—C27—N4—C26	0.5 (8)
N2—C12—C11—C10	0.3 (8)	O2—C7—O1—Ce1^i^	7.2 (6)
C14—C10—C11—C12	0.2 (7)	C3—C7—O1—Ce1^i^	−169.5 (2)
C9—C10—C11—C12	176.3 (5)	O1—C7—O2—Ce1	4.2 (8)
C6—C5—C8—O4	18.6 (7)	C3—C7—O2—Ce1	−179.1 (3)
C4—C5—C8—O4	−158.9 (4)	O6—Ce1—O2—C7	134.9 (5)
C6—C5—C8—O3	−162.3 (4)	O1^i^—Ce1—O2—C7	−12.8 (5)
C4—C5—C8—O3	20.1 (6)	O8^iii^—Ce1—O2—C7	−54.9 (5)
O6—C21—C17—C16	−166.5 (3)	O7^ii^—Ce1—O2—C7	−156.5 (5)
O7—C21—C17—C16	11.6 (5)	O9^iv^—Ce1—O2—C7	62.2 (5)
O6—C21—C17—C18	15.4 (5)	O1W—Ce1—O2—C7	127.6 (5)
O7—C21—C17—C18	−166.6 (3)	O2W—Ce1—O2—C7	−114.0 (5)
C18—C17—C16—C15	−2.2 (5)	O7—C21—O6—Ce1	7.3 (6)
C21—C17—C16—C15	179.6 (3)	C17—C21—O6—Ce1	−174.9 (2)
C17—C16—C15—C20	2.8 (5)	O1^i^—Ce1—O6—C21	−112.6 (4)
C17—C16—C15—N3	179.1 (3)	O8^iii^—Ce1—O6—C21	−70.1 (4)
C16—C15—C20—C19	−0.5 (5)	O7^ii^—Ce1—O6—C21	25.2 (3)
N3—C15—C20—C19	−176.6 (3)	O2—Ce1—O6—C21	90.9 (4)
C15—C20—C19—C18	−2.4 (5)	O9^iv^—Ce1—O6—C21	174.5 (4)
C15—C20—C19—C22	172.8 (3)	O1W—Ce1—O6—C21	98.3 (4)
C20—C19—C18—C17	3.0 (5)	O2W—Ce1—O6—C21	−42.3 (3)
C22—C19—C18—C17	−172.2 (3)	O6—C21—O7—Ce1^ii^	−112.8 (4)
C16—C17—C18—C19	−0.7 (5)	C17—C21—O7—Ce1^ii^	69.4 (5)
C21—C17—C18—C19	177.5 (3)	O9—C22—O8—Ce1^v^	85.9 (8)
O10—C23—C24—C25	−12.8 (7)	C19—C22—O8—Ce1^v^	−92.0 (8)
N3—C23—C24—C25	164.2 (4)	O8—C22—O9—Ce1^iv^	20.5 (5)
O10—C23—C24—C28	170.6 (5)	C19—C22—O9—Ce1^iv^	−161.5 (2)

Symmetry codes: (i) −*x*+1, −*y*+2, −*z*+1; (ii) −*x*+2, −*y*+2, −*z*+1; (iii) *x*−1, *y*+1, *z*; (iv) −*x*+2, −*y*+1, −*z*+1; (v) *x*+1, *y*−1, *z*.

### Hydrogen-bond geometry (Å, º)

**Table:**

*D*—H···*A*	*D*—H	H···*A*	*D*···*A*	*D*—H···*A*
O1*W*—H1*WA*···N2^vi^	0.85	2.21	2.756 (5)	122
O2*W*—H2*WB*···O1*W*^ii^	0.85	2.53	3.297 (5)	150
O2*W*—H2*WA*···O3*W*^vii^	0.85	2.07	2.728 (6)	133
O3*W*—H3*WA*···O10^viii^	0.85	2.06	2.856 (8)	157
O3*W*—H3*WB*···O5^ix^	0.85	1.92	2.742 (6)	161
O3—H3···N4^x^	0.82	1.81	2.583 (5)	156

Symmetry codes: (ii) −*x*+2, −*y*+2, −*z*+1; (vi) −*x*+1, −*y*+2, −*z*; (vii) *x*, *y*+1, *z*; (viii) *x*−1, *y*, *z*; (ix) −*x*+1, −*y*+1, −*z*+1; (x) −*x*+3, −*y*+2, −*z*+1.
